# ZnCo_2_O_4_‑Based Nanoparticle Sensor for 1‑Pentanol Detection

**DOI:** 10.1021/acsomega.5c08793

**Published:** 2026-01-30

**Authors:** Gabriela O. Gera, Gustavo S. M. dos Santos, André L. M Freitas, Diogo P. Volanti

**Affiliations:** † Federal University of ABC (UFABC), Center for Natural and Human Sciences, Santo André, SP 09210580, Brazil; ‡ Laboratory of Materials Sustainability (LabMatSus), São Paulo State University (UNESP), São José do Rio Preto, SP 15054-000, Brazil

## Abstract

When microbial volatile organic compounds (MVOCs) interact with humans, they can be harmful to health, causing irritation and discomfort. Additionally, they act as biomarkers for diseases. Despite the significance of this topic, there are still few detection tests available. This work explores the sensitive detection of MVOCs using semiconductor metal oxide (SMO). Therefore, this study aimed to evaluate the use of the monophase bimetallic structure ZnCo_2_O_4_ as a gas-sensing material for 1-pentanol detection, an MVOC that *Trichothecium roseum* and *Staphylococcus aureus* can produce. The study focused on analyzing the effect of a ZnCo_2_O_4_ nanostructure on enhancing the gas-sensing properties toward MVOCs detection. The material was prepared via microwave-assisted solvothermal processing and subsequently calcined at 300 °C to obtain a single-phase cubic ZnCo_2_O_4_. Characterization using XRD and FTIR revealed that the sample based on a bimetallic structure of ZnCo_2_O_4_ was successfully synthesized free of impurities, including the formation of undesired phases. Its performance as an MVOC sensor was analyzed for different volatiles, including 1-pentanol, 2-butanone, acetone, benzene, m-xylene, and toluene. The sensor demonstrated a higher selectivity for 1-pentanol, exhibiting the highest response of 83.6% to 1-pentanol (100 ppm) at 300 °C. Thus, this work presents an efficient method for producing single-phase cubic ZnCo_2_O_4_-based nanoparticles and demonstrates that this material exhibits enhanced sensing properties for the detection of 1-pentanol.

## Graphical abstract

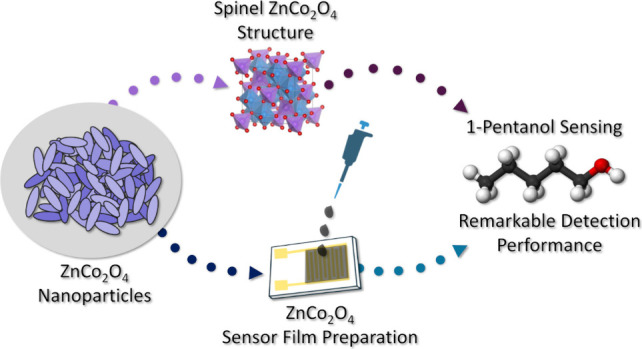

## 1 Introduction

During primary and secondary metabolism, microorganisms such as fungi and bacteria can produce microbial volatile organic compounds (MVOCs). These compounds are characterized by high vapor pressure, low molecular weight, and low boiling points, including different functional groups (alkenes, alcohols, ketones, terpenes, acids, and esters).^1,2^ Human exposure to these MVOCs can cause irritation and discomfort to the eyes and respiratory tract.^3,4^ Additionally, these compounds can act as biomarkers for different conditions, such as in the diagnosis of pulmonary bacterial infections and the detection of microbiological contamination in food.^5^ For example, 1-pentanol is an MVOC produced by the fungus *Trichothecium roseum*, a pathogen that infects fruit and causes pink rot, and by the bacterium *Staphylococcus aureus* in the contamination of natural fermentation.^6,7^ Therefore, developing detection techniques for 1-pentanol is of great interest to various fields due to the limited reported studies for this MVOC.

Although different technologies are used for VOC detection, standard platforms present important disadvantages for monitoring biomarkers such as 1-pentanol. Catalytic combustion sensors, for example, require high power consumption and are vulnerable to permanent poisoning by contaminants such as silicones.^8^ Electrochemical gas sensors detect gases via electrode reactions. They are sensitive, selective, and energy efficient. Nevertheless, they have a limited service life, and changes in humidity and temperature significantly affect their measurements.^9^ The thermal properties of gases influence heat transfer in the system, causing variations in response, a principle that underpins the operation of thermal conductivity sensors. However, these devices have limitations in terms of sensitivity and selectivity, particularly for detecting traces.^10^ To overcome these limitations, chemoresistive sensors stand out as a favorable alternative. They are compact and robust, with versatile applications and low production costs. Chemoresistive gas sensors can be constructed from various materials and composites, including semiconducting metal oxides (SMOs).^11^

In this scenario, gas sensors based on SMO have demonstrated promising materials capable of detecting volatile gases, even at low concentrations.^12,13^ Therefore, these sensors are a class of materials that present a functionalized metal to improve the electron transfer properties and chemoresistivity, affecting the detection efficiency of the sensor.^14–16^ SMO structures are easy to synthesize, abundant on Earth, environmentally friendly, chemically inert, and low-cost, with high adsorption of gas molecules on their surface.^17–19^ However, these materials typically exhibit poor selectivity and long response times, necessitating strategies to improve their sensing performance. ZnCo_2_O_4_ is a p-type SMO with a band gap of 2.6 eV and exhibits a spinel structure. Its band structure features a valence band (VB) associated with the O 2*p* orbitals, while the conduction bands (CBs) are related to the Co 3*d*–*eg,* and Co 3*d*–*t2g* levels. This configuration favors internal electronic transitions, contributing to a reduction in the recombination rate of electron–hole pairs generated by photonic excitation.^20,21^ In this way, it has been gaining prominence due to its potential applications. Kitchamsetti et al.^22^ synthesized MOF-derived ZnCo_2_O_4_ nanocages using the hydrothermal method to examine the photocatalytic elimination of pollutants, including methylene blue and rhodamine, achieving removal efficiencies of 84.3, 87.9, and 89.5% for CV, MB, and RhB dyes, respectively, after 180 min of irradiation. Hassan et al.^23^ utilized ZnCo_2_O_4_ incorporated with MOFs for the fabrication of electrodes for energy storage devices and the detection of monosodium glutamate. With a specific capacity of 984 C/g at 1.5 A/g, the ZnCo_2_O_4_@NiCo-MOF composite demonstrated better electrochemical performance than its constituent parts. The combined hybrid device (//AC) also produced a power density of 930 W/kg and an energy density of 92.4 Wh/kg.

Furthermore, zinc cobaltite has been gaining prominence as a gas detection sensor. Xu et al.^24^ synthesized ZnCo_2_O_4_ microtubes for detecting H_2_S, a highly toxic neurotoxic gas, at low temperatures. These hollow ZnCo_2_O_4_ microtubes calcined at 600 °C (ZCO-600) demonstrated excellent detection performance at 90 °C, achieving a detection limit of 50 ppb. Xiong et al.^25^ produced yolk–shell ZnCo_2_O_4_ microspheres obtained via a coprecipitation route, demonstrating superior sensing performance toward 500 ppm of acetone at 200 °C. The sensor’s response was 38.2, and its recovery and quick response times were 19 and 71 s, respectively. However, 1-pentanol monitoring is only briefly explored in the literature, particularly using bimetallic structures such as ZnCo_2_O_4_.

In this study, we described the synthesis of the bimetallic ZnCo_2_O_4_ using a microwave-assisted hydrothermal method with a mixture of green solvents (water and ethanol),^26^ followed by a calcination step, to evaluate its potential as an MVOC sensor. The sensing performance of ZnCo_2_O_4_ was investigated under various operating conditions, including temperature and relative humidity (RH), highlighting its enhanced sensitivity toward MVOC detection. The ZnCo_2_O_4_ sensor exhibited a higher response of 83.6% to 1-pentanol at 300 °C and remarkable performance under humid conditions.

## 2 Materials and Methods

### 2.1 Chemicals and Materials

All chemicals were purchased from Sigma-Aldrich, with a high degree of purity (≥99%), including zinc acetate dihydrate (Zn­(CH_3_COO)_2_·2H_2_O), cobalt­(II) acetate tetrahydrate (Co­(CH_3_COO)_2_·4H_2_O), and urea (CH_4_N_2_O). The MVOCs were prepared using high-purity reagents of m-xylene (≥99%), toluene (≥99.8%), benzene (≥99.8%), acetone (≥99.5%), 1-pentanol (≥99%), and 2-butanone (≥99.7%).

### 2.2 Synthesis of ZnCo_2_O_4_ Nanoparticles

The precursor of ZnCo_2_O_4_ was synthesized using an adapted procedure described by Minjie et al.^27^ First, 261.5 mg of Zn­(CH_3_COO)_2_·2H_2_O was dissolved in 30 mL of ethanol. Subsequently, 701.1 mg of Co­(CH_3_COO)_2_·4H_2_O and 1.0 g of urea were added to the previous solution. This mixture was then agitated for 10 min. . A polytetrafluoroethylene autoclave with a stainless-steel frame was filled with the resultant solution, and the solution was heated to 90 °C for 90 min using a microwave oven (2.45 GHz/800 W).^28^ The obtained precursor was centrifuged and washed with ethanol three times, and was dried at 60 °C overnight. The ZnCo_2_O_4_ sample was synthesized by calcining the precipitate at 300 °C with a heating rate of 5 °C/min for 10 min. The schematic representation of the synthesis is shown in Figure 1.

**Figure 1:**
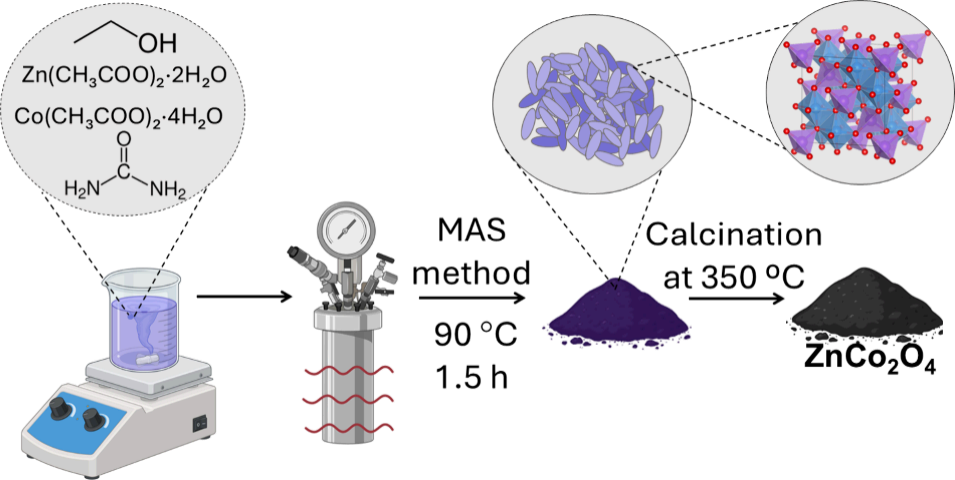
Schematic representation of the microwave-assisted solvothermal (MAS) synthesis process, followed by the precursor calcination to obtain the ZnCo_2_O_4_ nanoparticles.

### 2.3 Characterizations

Using a Rigaku MiniFlex 300 that had Cu Kα radiation (λ = 1.5418 Å) at 30 kV and 10 mA, X-ray diffraction (XRD) was used to analyze the crystalline phase. Measurements were carried out in the 2θ range between 5° and 80°, with a step size of 0.01° and a scan rate of 1° per minute. The specific surface area investigation of ZnCo_2_O_4_ was carried out by the Brunauer–Emmett–Teller (BET) method with a Micromeritics Gemini VII equipment, model 2390 t. Transmission electron microscopy (TEM) analyses were performed using a Thermo Fisher Scientific Talos F200X-G2 microscope, operated at 200 kV and equipped with an X-FEG field-emission gun. Image acquisition was carried out in bright-field (BF) and high-resolution transmission electron microscopy (HRTEM) modes. Additionally, compositional and atomic-scale contrast imaging were obtained using high-angle annular dark-field scanning transmission electron microscopy (HAADF-STEM) combined with energy-dispersive X-ray spectroscopy (EDS) for elemental mapping. We used a PerkinElmer Spectrum Two spectrophotometer to perform Fourier transform infrared (FTIR) spectroscopy, without heating the samples, operating in the 400–4000 cm^–1^ range. X-ray absorption spectroscopy (XAS) measurements were performed in total electron yield (TEY) and fluorescence yield (TFY) modes at the IPE beamline of Sirius, located at the Brazilian Synchrotron Light Laboratory (LNLS).

### 2.4 Gas Sensing Tests

To fabricate the sensor, 3.0 mg of the ZnCo_2_O_4_ powder was dispersed in 1 mL of isopropanol, and the mixture was subjected to an ultrasonic bath for 10 min to ensure homogeneous dispersion. 120 μL of the sample dispersion was deposited and coated onto alumina substrates with interdigitated gold electrode arrays measuring 0.1 mm × 5.0 mm and spaced 0.1 mm apart. The substrate was then heated at 300 °C for 1 h in a tubular oven to enhance sensor stability. The sensing response was measured from the change in electrical resistance. The resistance change was measured using a high-voltage source-measure device (Keithley SourceMeter 2400) at 5 V. The gas detection measurement scheme is shown in Figure S1. Subsequently, detection tests were conducted at different operating temperatures for 100 ppm of the following MVOCs: m-xylene, toluene, benzene, acetone, 1-pentanol, and 2-butanone. The concentration calculations for the prepared MVOCs tested were performed according to a previous study.^29^ The sensor measurements were conducted in 200, 250, 300, and 350 °C. Because ZnCo_2_O_4_ nanoparticles behave as a typical p-type semiconductor (low response), we report MVOCs responses as percentages, as in previous studies.^30^ Thus, the response (%) was obtained using the equation:

$$\mathrm{Response}\;(\%)=[(R_{\mathrm{MVOC}}/R_{\mathrm{Air}})-1]\times 100$$

where *R*_Air_ and *R*_MVOC_ are the electrical resistances of the sensor material in air and in the MVOC atmosphere, respectively, the response and recovery times were determined based on the time it took for the sensor to reach 90% of the maximum resistance change when interacting with the MVOC during adsorption and desorption. Details of the gas detection methodology are provided in the Supporting Information (SI). The I–V curves were measured in the range of −5 to 5 V, both at room temperature and at 300 °C. The tests were conducted using a Metrohm brand modular potentiostat/galvanostat, model Autolab.

## 3 Results and Discussion

The crystalline structure of the synthesized ZnCo_2_O_4_ was investigated by XRD analysis. Figure 2a shows the diffractogram of ZnCo_2_O_4_, which was indexed according to the cubic spinel ZnCo_2_O_4_ structure, JCPDS card No. 23-1390. No other peaks were observed, demonstrating the purity and single-phase properties of the synthesized material. Figure 2b shows the FTIR spectrum of ZnCo_2_O_4_. The bands observed at 555 and 657 cm^–1^ indicate the stretching vibrations of the M–O bond, confirming the octahedral and tetrahedral sites associated with Co–O and Zn–O, respectively.^31^ No other bands were observed. The surface properties of the material were analyzed using the BET method, as shown in Figure 2c, yielding a surface area of 14.52 m^2^/g. This analysis provides a more in-depth understanding of the material’s sensing properties, as gas diffusion reactions occur on its surface. The N_2_ isotherm obtained can be classified as a type IV isotherm with hysteresis, characteristic of mesoporous materials with pore sizes ranging from 2 to 50 nm, according to the IUPAC report.^32^ The increase in adsorbed volume at high relative pressures indicates pore-filling capacity and suggests the presence of interconnected mesopores. Figure 2d reveals that most pores are in the 2–30 nm range, with more concentrated peaks below 10 nm, confirming that ZnCo_2_O_4_ is predominantly mesoporous. The accumulated pore volume is approximately 0.04 cm^3^/g, indicating that the material is advantageous for adsorbing molecules.

**Figure 2:**
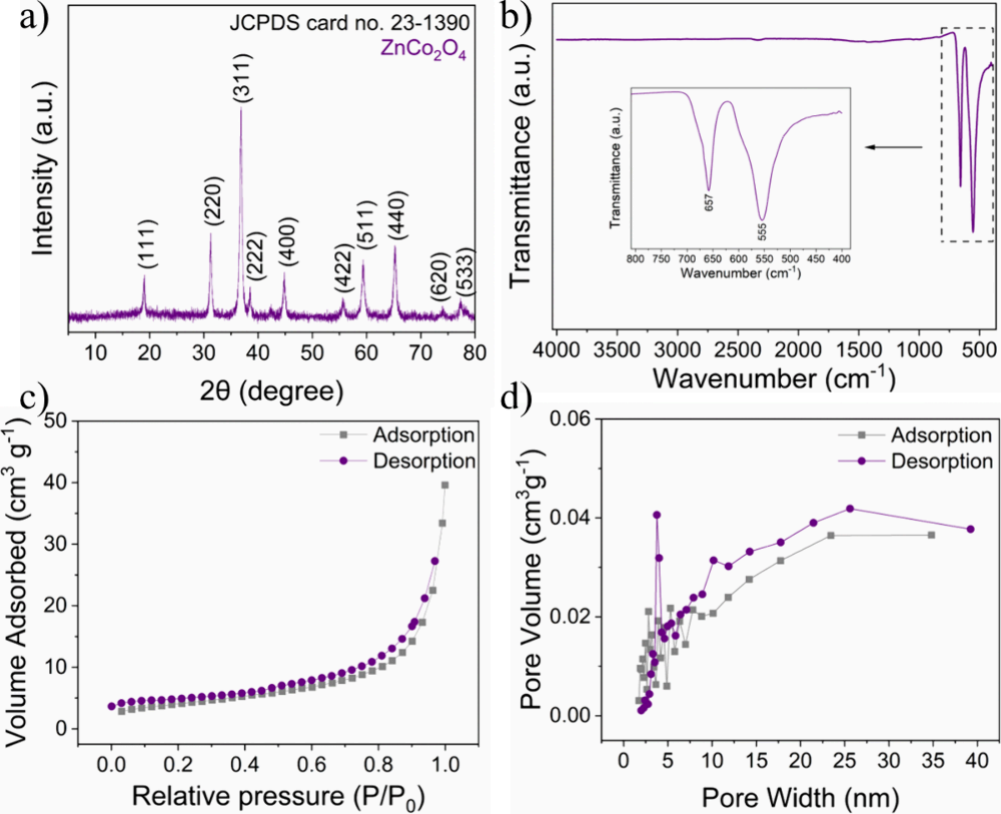
(a) XRD pattern, (b) FTIR spectrum, (c) isotherms by nitrogen adsorption and desorption, and (d) adsorption and desorption pore size distribution of ZnCo_2_O_4_.

To further analyze the microstructural features and morphological structure of the as-grown ZnCo_2_O_4_, SEM and TEM were conducted. SEM micrograph (Figure 3a) reveals a densely packed arrangement of agglomerated nanoparticles with irregular shapes and broad size distribution. This morphology suggests the formation of high surface area architecture, which is particularly advantageous for catalytic and gas-sensing applications.

**Figure 3:**
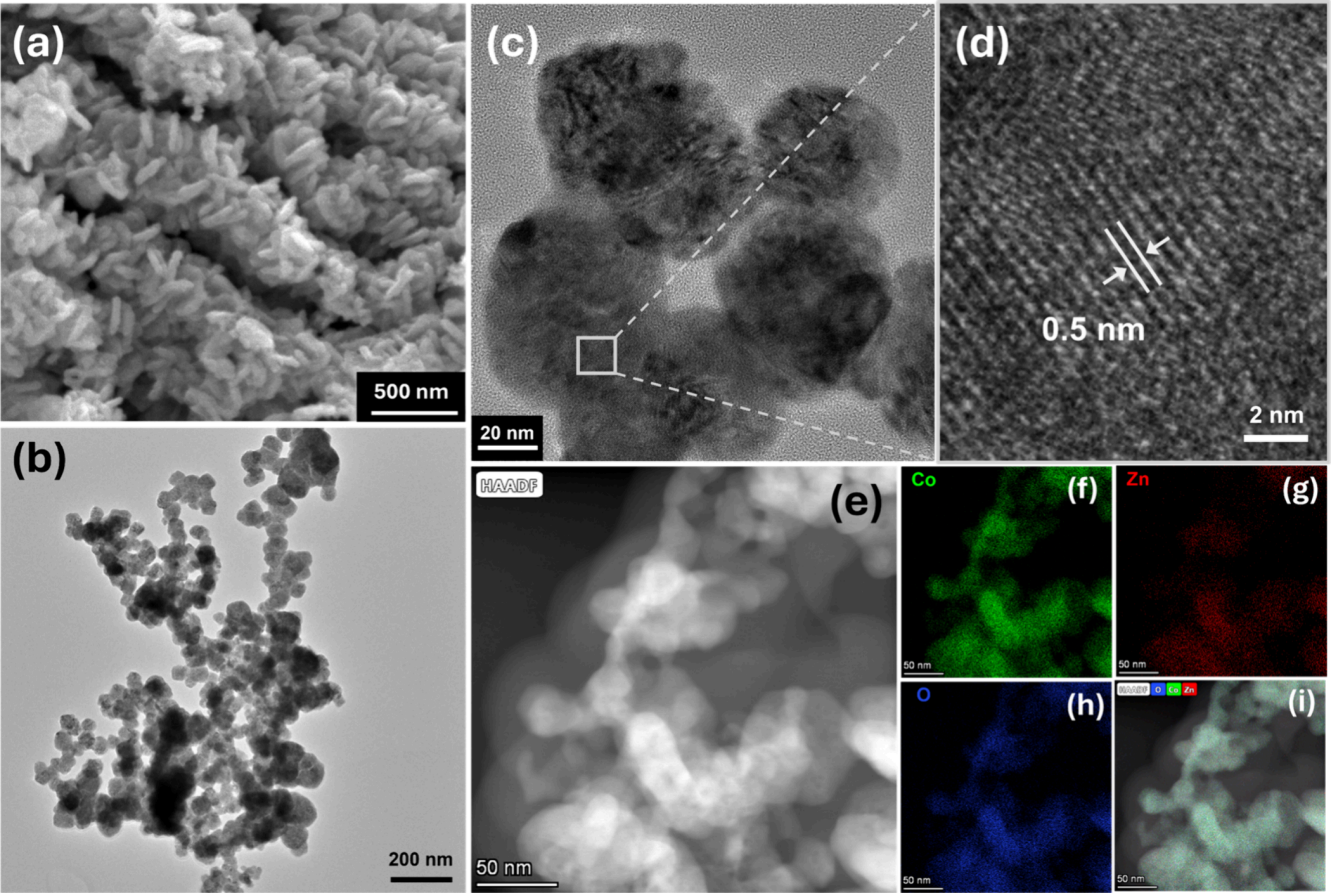
(a) SEM images of the synthesized ZnCo_2_O_4_ nanostructured assembly. (b) Low-magnification TEM image showing the overall morphology. (c) HRTEM image and (d) magnified selected region, highlighting the well-resolved lattice fringes and corresponding interplanar spacing. (e) HAADF-STEM image and corresponding EDS mapping for the selected area, showing the distribution of (f) Co, (g) Zn, (h) O, and (i) the merged EDS map.

To obtain further details on the morphological and structural characteristics of the synthesized ZnCo_2_O_4_ sample, TEM analysis was employed. As shown in Figure 3b, the material is composed of nearly spherical crystallites forming large aggregates. The contrast difference in TEM images suggests a high density of pores, which is consistent with the BET results. HRTEM images (Figure 3c,d) displayed a well-defined lattice fringe with an interplanar spacing of approximately 0.5 nm, indexed to the (111) crystallographic plane of the cubic spinel ZnCo_2_O_4_, which is in agreement with the XRD data presented earlier and is consistent with previous reports in the literature.^33–35^ Furthermore, the HAADF-STEM image (Figure 3e) and EDS elemental mapping (Figure 3f–h) confirm a uniform spatial distribution of Co, Zn, and O throughout the entire region. The overlay EDS map (Figure 3i) further supports the homogeneous colocalization of all constituent elements, indicating the successful formation of a well-mixed Co–Zn–O ternary oxide at the nanoscale, with no detectable phase segregation.

The EDS spectrum shown in Figure S2 provides a qualitative analysis of the elemental composition of the cobaltite sample ZnCo_2_O_4_. The first intense peak at low energy is attributed to carbon on the support grid; the other peaks in the spectrum correspond to the Kα and Lα lines of oxygen (O), cobalt (Co), and zinc (Zn). More intense Co peaks were observed relative to Zn, corresponding to its higher atomic ratio in the structure, in which two Co atoms are present for each Zn atom.

The electrical structure of the material was examined using XAS in TEY mode, which provides high surface sensitivity.^36^ The spectrum at the Co L_2,3_ edge of the material is shown in Figure 4a, which represents the electronic transitions from the occupied level of the Co 2*p* nucleus directly to the unoccupied states of Co 3*d*.^37,38^ In the L_3_ edge region, an intense peak at 781 eV (indicated as 2) stands out, characteristic of the presence of Co^3+^ ions.^39^ In contrast, a secondary peak (designated as 1) of lower intensity is identified at 779.5 eV, attributed to the Co^2+^ species. These results demonstrate the existence of two valence states of cobalt in the material.

**Figure 4:**
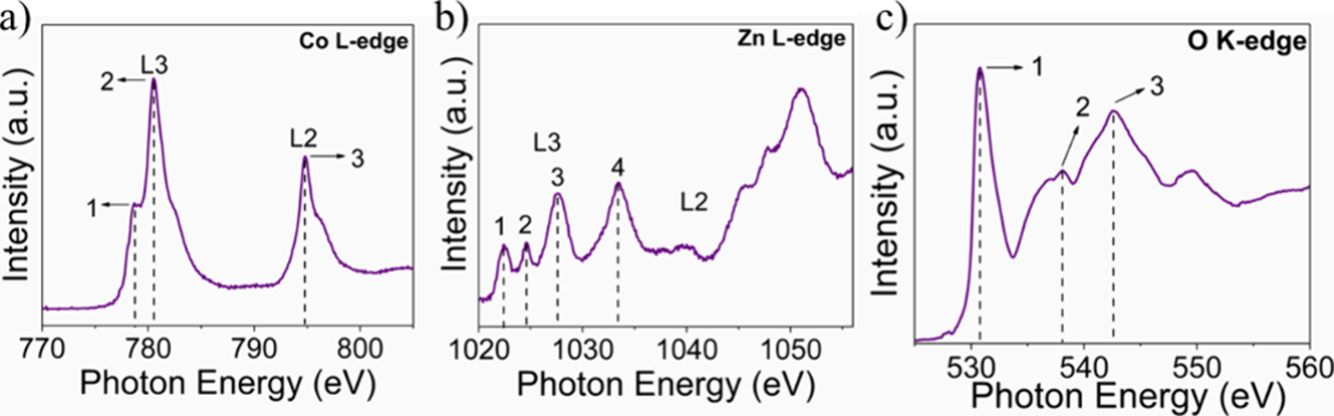
XAS spectra of ZnCo_2_O_4_ at the (a) Co L-edge, (b) Zn L-edge, and (c) O K-edge.

Figure 4b shows the Zn L spectrum. In the L-edge region, electronic transitions from the 2*p* level to the 4*s*, 4*p*, and 4*d* orbitals were investigated. The first peak (labeled as 1), located at 1024.3 eV, is related to these initial transitions. Subsequently, the other peaks in the regions between 1027.8 (peak 2), 1029.8 (peak 3), and 1034.1 eV (peak 4) are associated with transitions from the 2*p* to the 4*d* orbital.^40^

The K-edge XAS spectrum of oxygen is shown in Figure 4c and presents three peaks located at 530.2 eV (peak 1), 537.4 eV (peak 2), and 541.7 eV (peak 3). These peaks are related to the transitions of the O 1*s* orbital to unoccupied states with 2*p* character, hybridized with metal orbitals. Peak 1, located at 530.1 eV, indicates the presence of O species hybridized with low-spin Co^3+^ states.^41,42^

For the MVOC measurements, the operating temperatures were 200, 250, 300, and 350 °C. Below 200 °C, no significant sensing response were observed. Figure 5a shows the sensing responses of ZnCo_2_O_4_ to 100 ppm of six MVOCs (m-xylene, toluene, benzene, acetone, 1-pentanol, and 2-butanone) at the optimal operating temperature of 300 °C. The sample exhibited the highest response to 1-pentanol, with a response value of 83.6%. Figure 5b summarizes the responses of the sample to 1-pentanol at different temperatures. A higher response of 83.6% was observed at 300 °C and decreased as the temperature increased, confirming the optimal operating temperature. Figure S3 presents the responses of ZnCo_2_O_4_ at 100 ppm of different MVOCs as a function of temperature, indicating that the most suitable operating temperature for most of the tested MVOCs is 300 °C. The I–V curves, shown in Figure S4 in the support information, confirm an ohmic contact at 300 °C, ensuring a stable baseline for these sensing measurements.

**Figure 5:**
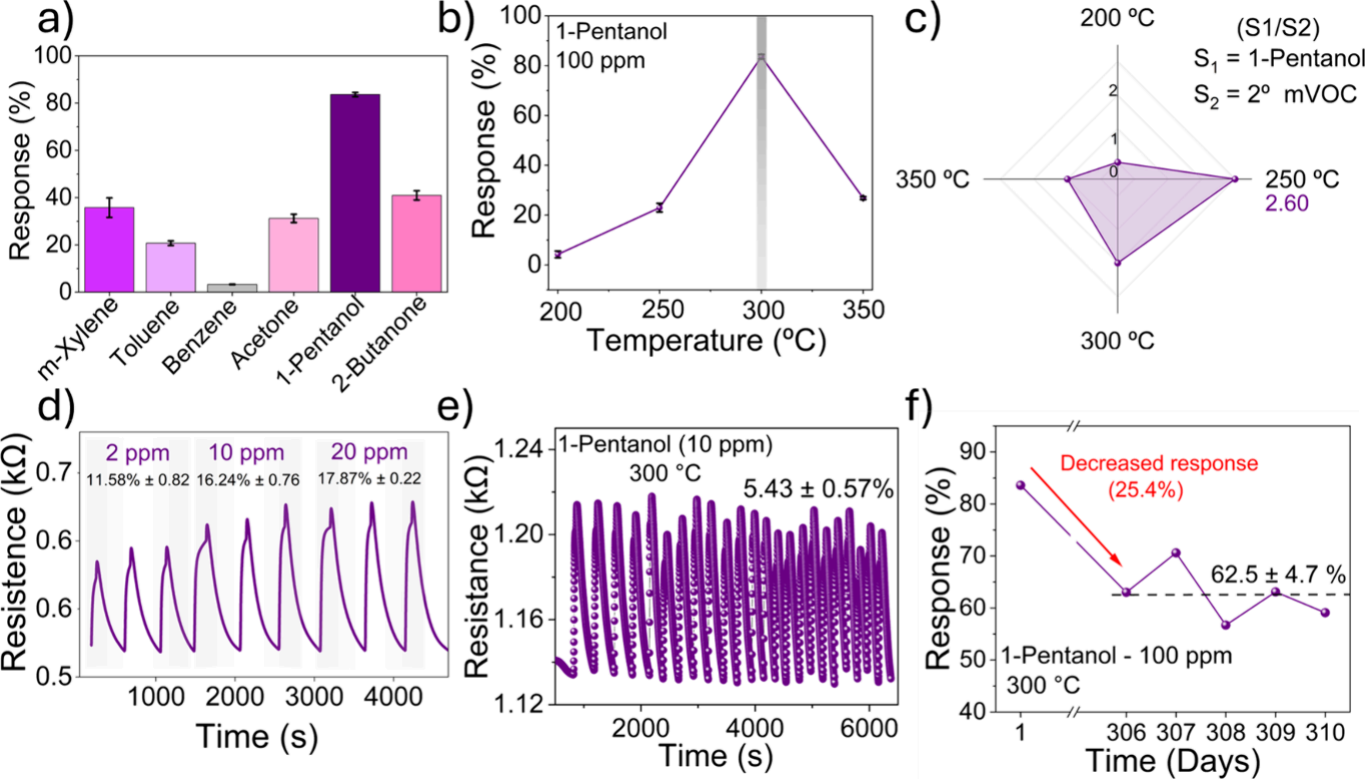
(a) Sensing responses of ZnCo_2_O_4_ at 300 °C to 100 ppm of six MVOCs: m-xylene, toluene, benzene, acetone, 1-pentanol, and 2-butanone; (b) sensor responses to 1-pentanol as a function of temperature; (c) radar graph of the selectivity index to 1-pentanol at different temperatures (200, 250, 300, and 350 °C); (d) responses to varying concentrations of 1-pentanol; (e) repeatability over 23 exposure cycles to 10 ppm of 1-pentanol at 300 °C; and (f) sensor response stability on day 1 and after 306 days.

The selectivity index (SI) of the ZnCo_2_O_4_ sensor was determined from the relationship between the responses of the target gas (1-pentanol) and the interfering gas (2-butanone), as shown in Figure 5c. The sensor exhibited a higher selectivity value of 2.6 at 250 °C. While the selectivity index (SI) at the optimum operating temperature was 2.07 against 2-butanone, the MVOC showed the second-highest response. The ZnCo_2_O_4_ responses to different concentrations (ppm) of 1-pentanol at 300 °C under a dry air atmosphere are shown in Figure 5d, exhibiting the MVOC detection in low concentrations, such as 2 and 20 ppm, with response values of around 11.55, 16.24, and 17.87%. Nevertheless, linear regression analysis yielded a theoretical limit of detection (LOD) of 0.83 ppb with *R* ^2^ = 0.9822, highlighting the high sensitivity and efficiency of the ZnCo_2_O_4_ sensor (Figure S5). The repeatability of the sensor was evaluated using 10 ppm of 1-pentanol at 300 °C under dry air conditions (Figure 5e). Over 23 consecutive cycles, the sensor maintained a stable and consistent response of 5.43 ± 0.57%, demonstrating remarkable reversibility. The long-term stability of the sensor was evaluated after 306 days to verify the repeatability of responses to 1-pentanol at 300 °C. As illustrated in Figure 5f, on the first day of measurement, the response was 83.6%. After 306 days, a 25.4% reduction in response was observed, resulting in a 63% response rate. This value remained stable throughout the subsequent 5 days of testing, with an average response of 62.5 ± 4.7%, indicating good reproducibility of the material after several months.

The response/recovery times of the ZnCo_2_O_4_ sensor to 100 ppm of 1-pentanol at 300 °C under different RH values were evaluated. In a dry atmosphere (Figure 6a), the sensor exhibited a response time of 4.4 s and a recovery time of 416.2 s. As the RH increased to 23 and 39% (Figure 6b,c), the response times increased to 11.0 s. In contrast, the recovery times increased to 509.6 and 679.3 s, respectively, which may be due to competition between water vapor and the analyte for active sites on the sensor surface. At higher humidities of 63 and 78% RH (Figure 6d,e), the response time decreased to 8.4 s, which may be due to a water layer that facilitates analyte diffusion. However, recovery times continued to increase (694.9 and 715.6 s, respectively), likely due to the difficulty in desorbing 1-pentanol in humidity-saturated environments. Although the sensor’s recovery time increases with humidity, some reported methods aim to optimize it by using UV light irradiation. When exposed to light, the sensor modifies its surface electrical characteristics, exciting charge carriers and generating greater interaction with gas molecules.^43^ Another technique used to optimize the sensor’s recovery time is pulse heating, since heat provides the necessary activation energy to accelerate the desorption of adsorbed gas molecules. These strategies prove efficient for future studies of this material.^44^ Despite the variations in response and recovery times, the graph in Figure 5f demonstrates that the sensor responses remained stable at different humidity levels, with an average of 58.5 ± 1.1%, demonstrating robustness and remarkable humidity resistance, which is essential for real-world applications.

**Figure 6:**
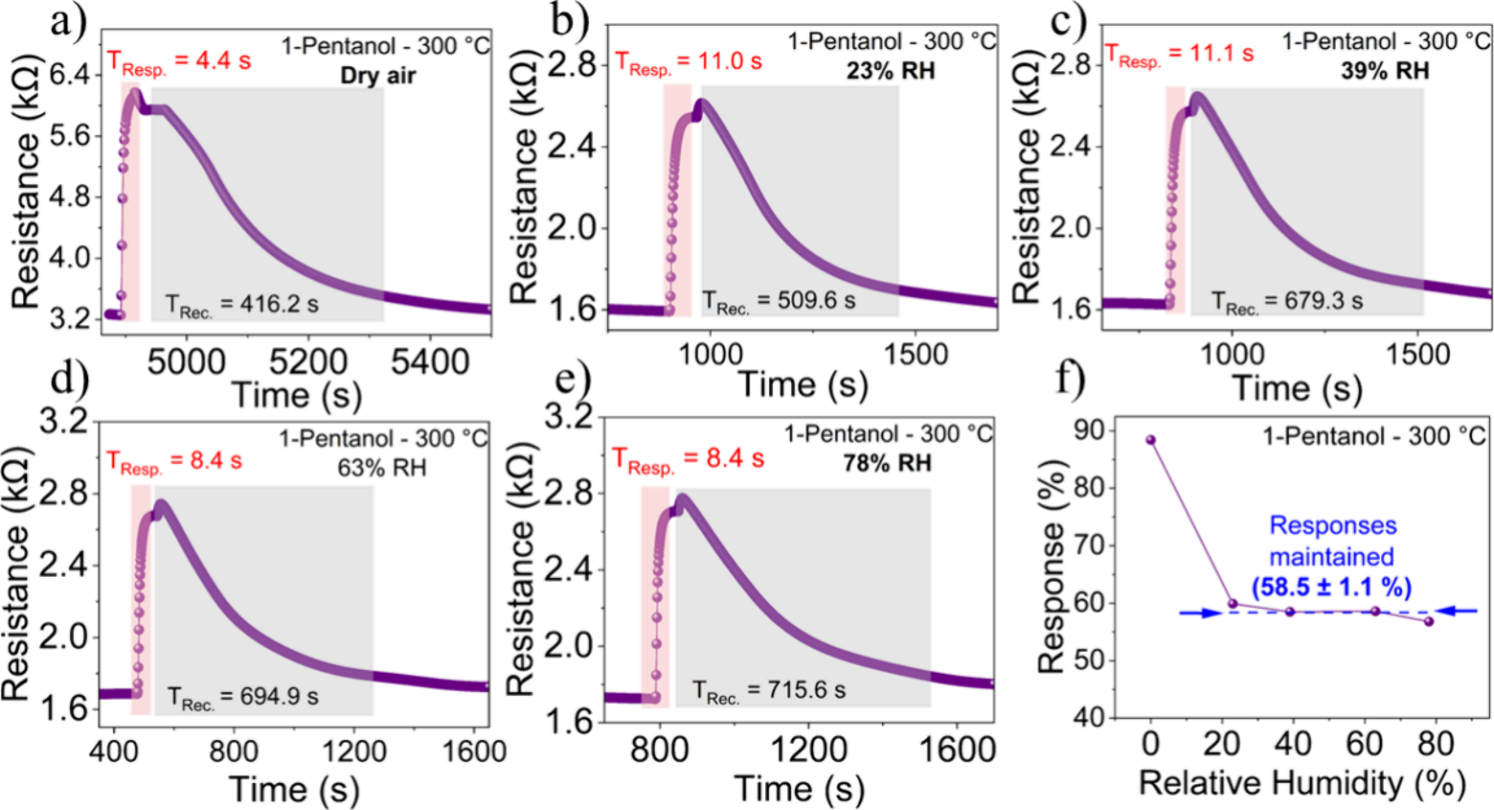
Response and recovery time curves for the ZnCo_2_O_4_ sensor to 100 ppm of 1-pentanol at 300 °C under different RH conditions: (a) dry air, (b) 23% RH, (c) 39% RH, (d) 63% RH, (e) 78% RH, and (f) Response as a function of RH.

An overview of the main performance characteristics of the ZnCo_2_O_4_ sensor compared to other sensors reported in the literature for *n*-pentanol detection is presented in Table 1. Furthermore, it is worth noting that there are few studies focused on detecting *n*-pentanol. Although the response of our sensor is moderately low, the response and recovery times indicated superior performance compared to other materials. The ZnCo_2_O_4_ sensor exhibited a response time of 4.4 s. In contrast, competing sensors either exhibited extremely high recovery times (above 1443 s) or, even if they had lower recovery times, they suffered from significantly longer response times. Therefore, the developed sensor showed balanced and good response and recovery times for 1-pentanol detection.

**Table 1:** Comparison of the Sensing Capabilities of Several Materials for 1-Pentanol

**Material**	**Semiconductor type**	**Working T (°C)**	**Conc.(ppm)**	**Response** ^a^	**Response/Recovery time (s)**	**Ref.**
ZnO nanotubes	n	252	100	221	19/56	^45^
RuCu-SnO_2_	n	200	50	217.83	20/146	^46^
In_2_O_3_ microrods	n	350	100	390	2/1443	^47^
Nanostruct. ZnO-450	n	400	100	2680	1.08/4737	^48^
Nanostruct. ZnO-600	n	400	100	2205.5	0.60/3091	^48^
Nanostruct. ZnO-800	n	400	100	231.8	0.94/2883	^48^
ZnCo_2_O_4_ nanoparticles	p	300	100	1.83^b^	4.4/416.2	This work

a (*R*_air_/*R*_1‑pentanol_) for n-type and (*R*_1‑pentanol_/ *R*_air_) for p-type materials.

b Equivalent response of 83.6%.

### 3.1 Gas Sensing Mechanism

The detection mechanism of semiconductor metal oxides is based on changes in resistance resulting from the chemical adsorption of oxygen molecules at the material’s surface, generating negatively charged oxygen species (O^–^, O_2_^–^, O^2–^). These generated species depend on the operating temperature.^49^ The optimal operating temperature of our sensor (ZnCo_2_O_4_) is 300 °C, indicating that the predominant species is O ^–^.^50^

$$O_{2\left(\mathrm{ads}\right)}+e^{-}⇌{{O_{2}}^{-}}_{\left(\mathrm{ads}\right)}$$ 1

$${{O_{2}}^{-}}_{\left(\mathrm{ads}\right)}+e^{-}⇌2{O^{-}}_{\left(\mathrm{ads}\right)}$$ 2

Charge transport in p-type ZnCo_2_O_4_ nanoparticles occurs primarily via holes (h^+^) in the valence band (*E*_v_), and the sensing mechanism involves surface oxygen adsorption in air, which attracts electrons from the conduction band (*E*_c_). When a reducing gas, in this case 1-pentanol, reaches the surface of the ZnCo_2_O_4_ nanoparticles, it reacts with the chemisorbed oxygen. During this reaction, electrons are released and recombine with holes in the cobaltite, reducing the thickness of the hole-accumulation layer and the potential barrier (ΔΦ). As a result, the concentration of majority carriers decreases, reducing electrical conductivity and, consequently, increasing the sensor’s resistance due to electron–hole recombination.^51,52^ The illustrative scheme of the detection of 1-pentanol is shown in Figure 7.

**Figure 7:**
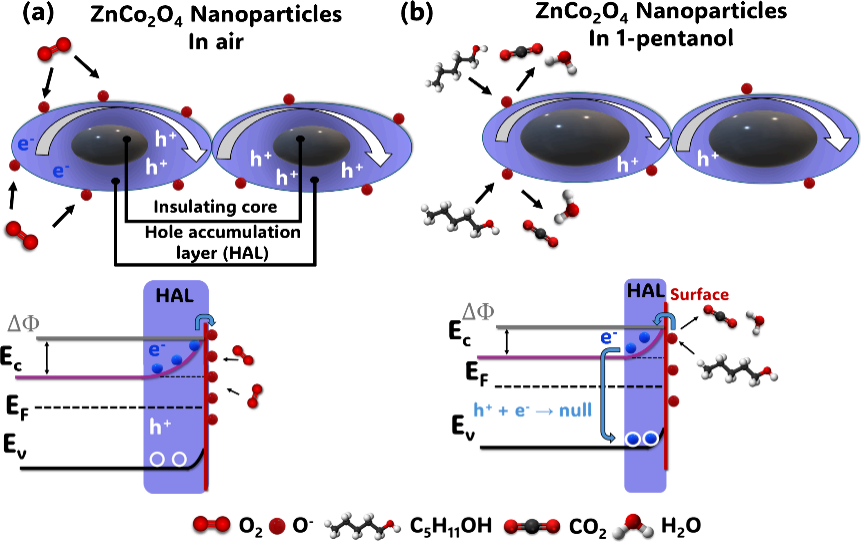
Scheme for the proposed detection mechanism using ZnCo_2_O_4_ nanoparticles in (a) air and (b) 1-pentanol.

## 4 Conclusions

In summary, the p-type gas sensor composed of ZnCo_2_O_4_ nanoparticles synthesized by the MAS method, followed by calcination, demonstrated excellent performance in detecting 1-pentanol, with a higher response of 83.6% to 100 ppm at 300 °C and maintained noticeable performance under various RH conditions. Furthermore, it demonstrated a short response time of 4.4 s for 1-pentanol in dry air and 8.4 s at higher humidity levels. These parameters highlight the material as an efficient sensor under complex humidity conditions, and such results demonstrate promising potential for future applications yet to be explored. Furthermore, the material demonstrates high potential for detecting volatile organic compounds, which serve as biomarkers of disease and indicators of food degradation, such as fruit spoilage.

## Supplementary Material



## Notes

Schematic of the gas-sensing measurement setup; description of the procedure for sensor baseline stabilization and calculation of MVOC concentration; energy-dispersive X-ray spectroscopy (EDS) spectrum with elemental quantification of the synthesized sample; graphic with sensor responses to 100 ppm of different MVOCs in dry air as a function of temperature; and I–V curves of the ZnCo2O4 sensor measured at 300 °C and room temperature, including an analysis of the conduction behavior (PDF)
