# Supplementary material for: ZnCo2O4‑Based Nanoparticle Sensor for 1‑Pentanol Detection
Source: ACS Omega. 2026 Jan 30;11(5):7566–73. doi: 10.1021/acsomega.5c08793 (PMC12902984; doi:10.1021/acsomega.5c08793)
Supplement: Supplementary file 1 [file ao5c08793_si_001.pdf]

Supporting Information

$ZnCo_2O_4$-based Nanoparticles Sensor for 1-Pentanol Detection

Gabriela O. Gera,[a] Gustavo S. M. dos Santos,[b] André L.M Freitas, [a]Diogo P. Volanti*[b]

[a] *Federal University of ABC (UFABC), Center for Natural and Human Sciences, 09210580, Santo André, SP, Brazil.*

b Laboratory of Materials Sustainability (LabMatSus), São Paulo State University (UNESP),15054-000, São José do Rio Preto, SP, Brazil.

**Corresponding author. E-mail address: diogo.volanti@unesp.br (Diogo P. Volanti)*

Procedure for sensor baseline stabilization and calculation of MVOC concentration

For baseline stabilization, the sensor was exposed to air flow for 1 h at 300 °C. The temperature was monitored with a thermocouple inserted into the tubular furnace (Fig. S1), as in your previous work.[1] To measure the change in electrical resistance, sensors with different MVOC concentrations were initially prepared in glass vials containing only dry air, as described by the following equation.

$$C = \frac{22.4p\,TVs}{273MV}\,x\,1000$$

C = MVOC concentration (ppm),

p = liquid density (g mL^{-1}),

T = temperature during testing (K),

Vs = volume of the MVOC (mL),

M = molecular weight of the MVOC (g mol^{-1})

V = volume of the test chamber (tube) = 754 mL.

First, the MVOC were fully evaporated inside the closed vial, and in follow, a calculated volume of the MVOC vapor was collected using a syringe. Subsequently, the MVOC vapor (contained within the syringe) was introduced into the test chamber to achieve the desired concentration via dilution. After the sensor signal stabilized, a pure air flow of (250 mL/min) was used to purge the tube oven atmosphere.

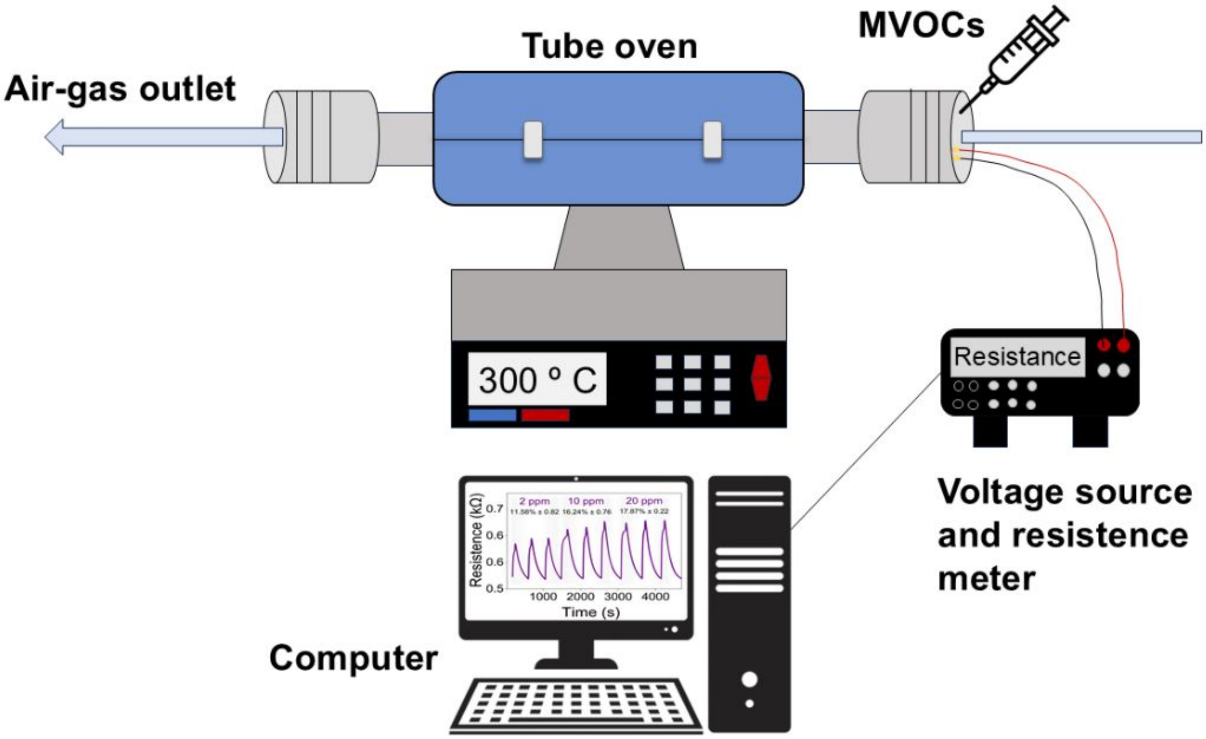

Fig. S1. Schematic representation of the sensor measurement system.

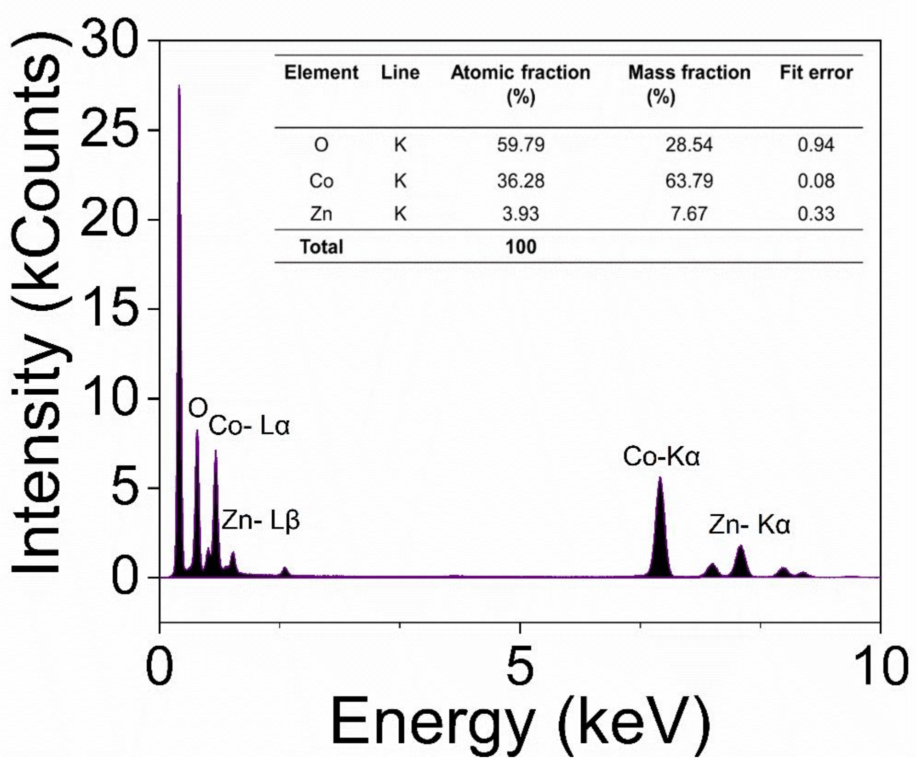

Element	Line	Atomic fraction (%)	Mass fraction (%)	Fit error
O	K	59.79	28.54	0.94
Co	K	36.28	63.79	0.08
Zn	K	3.93	7.67	0.33
**Total**		**100**		

Fig. S2. Energy-dispersive X-ray spectroscopy (EDS) spectrum.

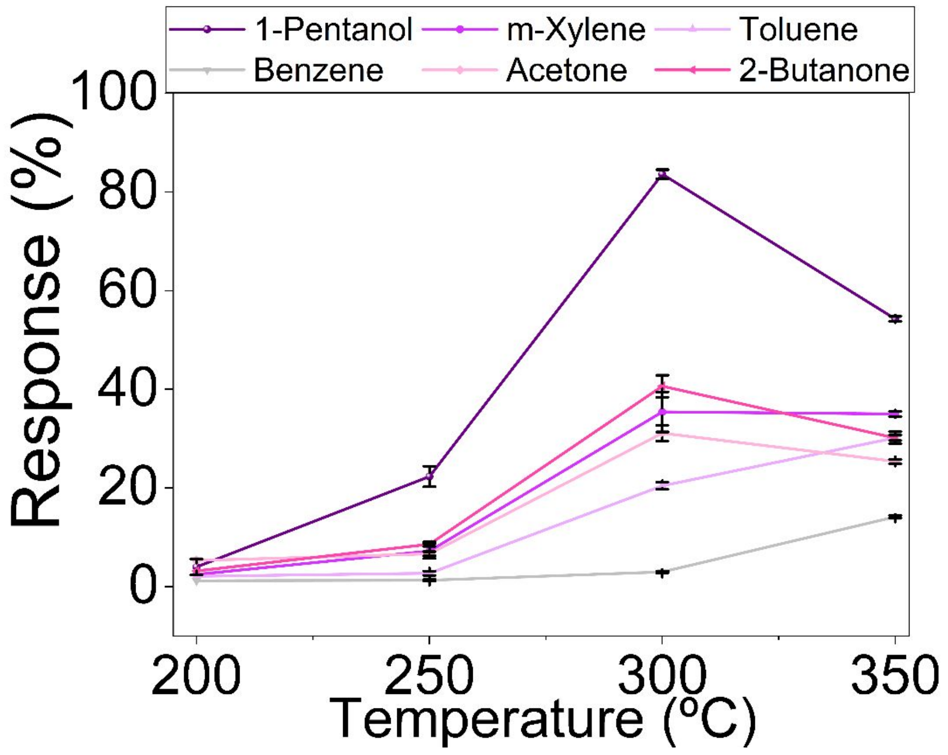

Fig. S3. Responses to 100 ppm of different MVOCs in dry air as a function of temperature for $ZnCo_2O_4$.

I-V curves of the $ZnCo_2O_4$ and analysis of the conduction behavior

The sensor exhibits an activation potential of approximately 0 to 0.5 V, indicating that the conduction process is not ohmic over the analyzed potential range (Fig. S4). However, above 1.0 V, a region of approximately linear current-potential response is observed, which coincides with the sensor's practical operating range. In this regime, conduction occurs after overcoming the initial barrier, resulting in charge transport that varies in proportion to the applied potential. Thus, although the overall behavior is not strictly ohmic, the device can be considered to operate almost linearly in the region of interest, including the fixed operating potential of 5 V.

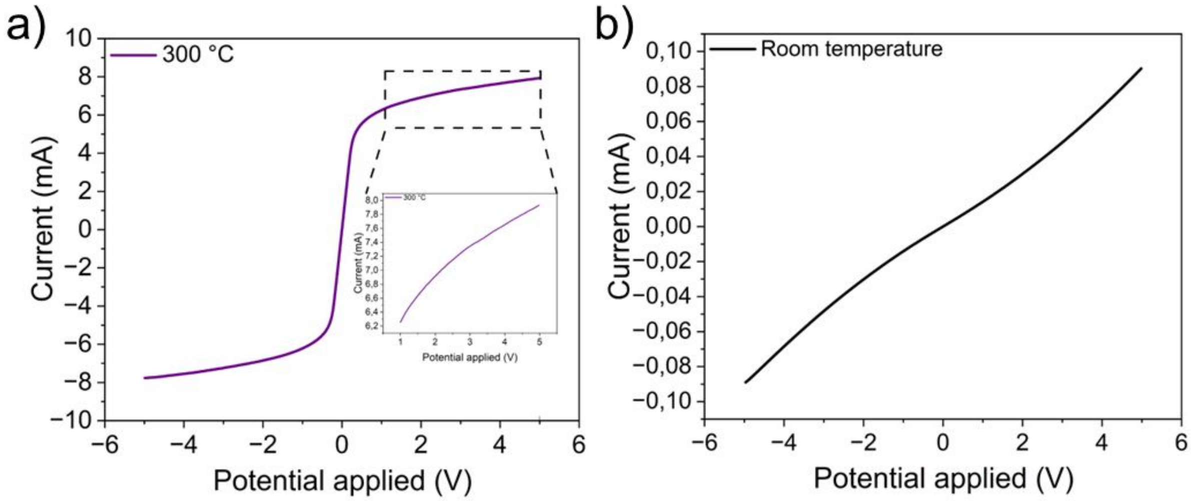

Fig. S4. I-V curves of the $ZnCo_2O_4$ sensor measured **(a)** at 300 °C, with detail of the 0-5V region, and **(b)** at room temperature.

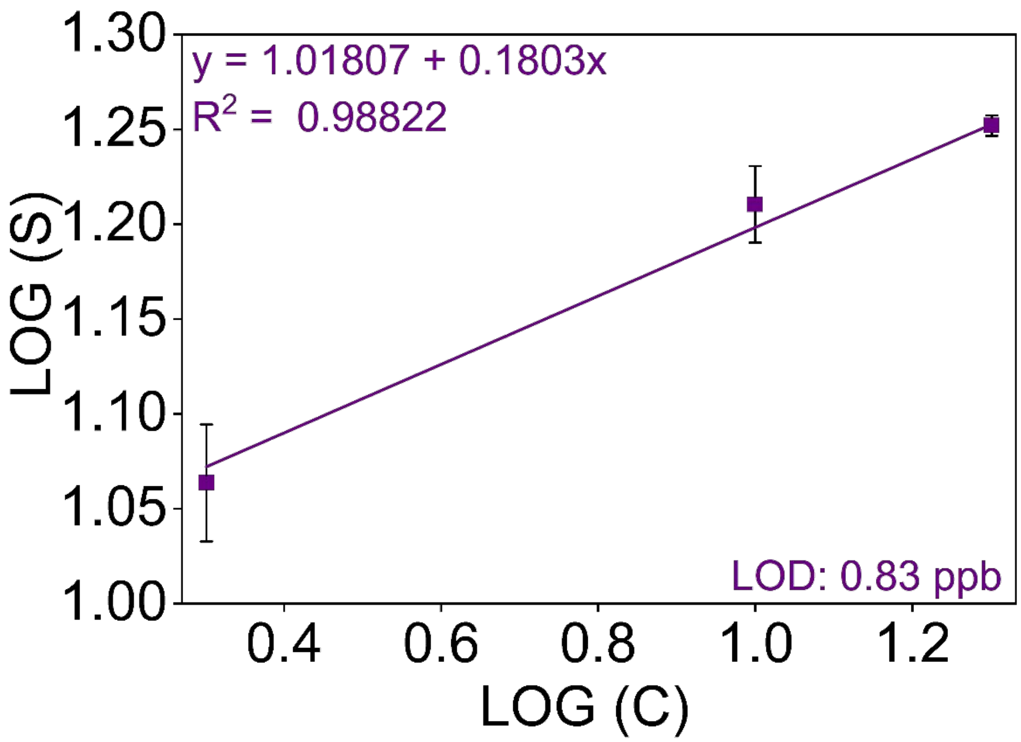

Fig. S5. The logarithm of the 1-pentanol response (S) versus the logarithm of the 1-pentanol concentration (C).

Reference

(1) Sanghikian Marques dos Santos, G.; Theodoro, R. dos S.; Gera, G. O.; Micheli Perfecto, T.; Paschoalini Volanti, D. Ethyl Acetate Detection Using Mixed-Phase In_2O_3 Nanorods. *ACS Appl Nano Mater* **2025**. https://doi.org/10.1021/acsanm.5c01487.
